# Body height, body weight, BMI, and nutritional status among male adolescents in Montenegro: a nationwide archival study from 1979 to 1987

**DOI:** 10.3389/fpubh.2026.1767488

**Published:** 2026-03-03

**Authors:** Stevo Popovic, Jovan Gardasevic, Srdja Martinovic, Bojan Masanovic, Radenko M. Matic

**Affiliations:** 1 Faculty for Sport and Physical Education, University of Montenegro, Niksic, Montenegro; 2 Western Balkan Sport Innovation Lab, Podgorica, Montenegro; 3 Department of Physical Education, Korea University, Seoul, Republic of Korea; 4 Faculty of Law, University of Montenegro, Podgorica, Montenegro; 5 Faculty of Sport and Physical Education, University of Novi Sad, Novi Sad, Serbia

**Keywords:** adolescence, anthropometry, military conscripts, Montenegro, nutritional status, secular trends

## Abstract

**Introduction:**

Monitoring anthropometric characteristics such as body height and body weight, as well as body mass index (BMI) is central to understanding biological develop¬ment and population health. Montenegro is known for exceptionally tall stature, which is also reflected in other mentioned variables, yet historical national-level data on adolescent growth during the second half of the 20th century have been limited. The aim of this study was to examine secular trends in body height, body weight, BMI, and nutritional status among male adolescents in Montenegro using nationwide archival data from mandatory military medical examinations con¬ducted between 1979 and 1987.

**Methods:**

This retrospective cross-sectional study included 83,274 male adolescents born between 1961 and 1969. Data were obtained from digitized archival medical records collected during standardized pre-recruitment examinations in the former Yugoslav People’s Army. The mean age at exami¬nation was 18.06 ± 0.52 years (range: 16.5–25.9), supporting the application of adult BMI classification criteria. Body height, body weight, and BMI were analyzed by birth cohort using analysis of variance and linear regression models, while trends in nutritional status categories were assessed using chi-square tests for trend.

**Results:**

The mean body height of the total sample was 176.76 ± 6.91 cm, confirm¬ing that Montenegrin adolescents belonged to one of the tallest populations in Europe during the study period. Although statistically significant differences across cohorts were observed due to the large sample size, effect sizes and regression analyses indicated no meaningful secular changes in body height, body weight, or BMI. Nutritional status analysis showed that 85.9% of participants had normal BMI, while the prevalence of overweight (7.2%) and obesity (0.6%) was low. A modest but statistically significant increase in overweight and obesity prevalence across cohorts was identified.

**Discussion:**

This study provides the first compre¬hensive national overview of anthropometric characteristics and nutritional status of male adolescents in Montenegro during the late 20th century. By establishing a relevant historical baseline based on standardized nationwide archival data, the findings provide an essential reference for future analyses of long-term secular trends and contemporary public health comparisons.

## Introduction

Monitoring anthropometric characteristics such as body height and body weight, as well as body mass index (BMI) represents a central topic in biological anthropology and public health. Long-term population measurements of these indicators, particularly within military systems, provide valuable insights into biological development, living conditions, and population health across generations (1, 2). Numerous studies have demonstrated a general increase in average adult body height during the 20th century across most industrialized regions, largely attributed to improvements in nutrition, healthcare, and socioeconomic conditions (3, 4). Consequently, body height is widely considered an integrated biological indicator of population health and environmental conditions during growth and development (5).

Montenegro is part of the Dinaric Alps region, which has long been recognized in anthropological literature as the region with the tallest populations worldwide (6–8). Contemporary studies consistently confirm the exceptional stature of Montenegrin adolescents and young adults, positioning them among the tallest globally (9). At the same time, recent national and international reports indicate growing concerns related to increasing prevalence of overweight and obesity, as well as unfavorable patterns of physical activity and dietary behavior among younger generations (10–13).

Despite these findings, systematic historical monitoring of anthropometric characteristics in Montenegro has been limited. Available evidence for the second half of the 20th century remains fragmented and largely confined to local or regional studies (14–18), preventing reliable assessment of long-term secular trends at the national level (19, 20). This lack of comprehensive historical data represents a major obstacle for understanding the dynamics of biological development and nutritional changes across generations.

Military medical records represent one of the most reliable sources of historical anthropometric data, as examinations were conducted using standardized procedures and included nearly the entire male adolescent population. In Montenegro, recent collaboration between the Ministry of Defense and the University of Montenegro enabled the digitization of extensive archival records of conscripts from the former Yugoslav People’s Army, creating a unique nationwide dataset. Initial analyses based on selected municipalities suggested relatively stable trends in body height, body weight, and BMI during the 1980s, although changes in nutritional status distribution were observed (17). However, the extent to which these findings reflect nationwide patterns has remained unclear. Given the well-documented socioeconomic and demographic diversity across Montenegro, as well as differences in lifestyle and health indicators between regions, the use of a comprehensive national sample is essential for reliable assessment of historical trends from the national perspective.

The aim of the present study was to examine secular trends in body height, body weight, BMI, and nutritional status among male adolescents in Montenegro who underwent mandatory military medical examinations between 1979 and 1987, using a nationwide archival dataset. By establishing a relevant historical baseline for late adolescence, this study provides an essential reference framework for interpreting long-term changes in anthropometric characteristics and nutritional status within the Montenegrin population.

## Materials and methods

This study employed a retrospective cross-sectional design and was based on data collected during mandatory medical examinations conducted prior to military recruitment in the former Yugoslav People’s Army. As part of a national project aimed at the digitization of archival medical documentation, individual records of male conscripts from all municipalities of Montenegro were consolidated into a single database. Access to the archival records and their scientific use were formally approved by the Ministry of Defense of Montenegro.

Of the initially digitized records (*N* = 85,168), a total of 1,894 (2.2%) were excluded due to missing key variables (body height, body weight, year of birth, or year of examination), internal inconsistencies, or incomplete archival entries, yielding a final analytical sample of 83,274 participants. No additional arbitrary anthropometric cut-off thresholds were imposed during the digitization process, as all measurements had originally been collected and verified by trained military medical personnel using standardized procedures within the Yugoslav People’s Army medical system. These official medical records underwent routine clinical verification at the time of examination and were therefore considered highly reliable. During digitization, all entries were nevertheless subjected to logical and internal consistency checks, including verification of chronological coherence between year of birth and year of examination, recalculation of BMI from recorded height and weight, and screening for incomplete, duplicate, or mutually inconsistent entries. Any records identified as logically inconsistent or incomplete were excluded from the final analytical dataset. To further ensure transcription accuracy, a random subsample of digitized records was cross-checked against the original archival medical documents. This verification step confirmed a high level of agreement between the digitized database and the original paper records, supporting the reliability of the digitization process and the integrity of the final analytical dataset. The analyzed sample comprised 83,274 male adolescents born between 1961 and 1969 who underwent standardized medical examinations for military service. Given the large sample size and broad geographical coverage, the data represent a comprehensive cross-section of the male population of Montenegro at that time.

The mean age at examination was 18.06 ± 0.52 years (range: 16.53–25.96). The majority of participants were examined at approximately 18 years of age in accordance with standard pre-recruitment procedures. The presence of older examinees in the dataset primarily reflects postponed military service due to higher education enrollment, temporary medical deferment, or other administrative reasons, as well as re-examinations conducted within the conscription system. Such age variability was a common and expected feature of the mandatory conscription procedures of the former Yugoslav People’s Army and could not be influenced by the researchers, as the study relied exclusively on archival administrative records. Despite this variation, the age distribution remained strongly centered around late adolescence, and the use of adult BMI classification criteria was considered appropriate and consistent with previous research based on military conscript populations.

Measurements were conducted in military health centers throughout Montenegro during the period from April 20, 1979, to July 1, 1987. All examinations were performed by trained physicians and medical personnel in accordance with a unified and standardized protocol applied across all recruitment centers. Participants were measured barefoot and wearing light underwear. Body height was measured using a medical stadiometer and recorded to the nearest centimeter, while body weight was assessed using calibrated medical scales and recorded to the nearest kilogram. BMI was calculated as body weight in kilograms divided by the square of body height in meters (kg/m^2^). Nutritional status was defined according to internationally accepted BMI categories: underweight (BMI < 18.5 kg/m^2^), normal weight (BMI 18.5–24.9 kg/m^2^), overweight (BMI 25.0–29.9 kg/m^2^), and obesity (BMI ≥ 30.0 kg/m^2^), in accordance with World Health Organization (WHO) classification criteria (21).

Data were processed using the SPSS 20.0 statistical software package (Chicago, IL, USA). Descriptive statistics were calculated for each birth cohort from 1961 to 1969 and expressed as means and standard deviations for all variables. Differences between birth cohorts were examined using one-way analysis of variance (ANOVA), with birth year treated as a categorical independent variable. Effect sizes were interpreted alongside *p*-values in order to assess the practical significance of observed differences. Secular trends across successive birth cohorts were additionally assessed using linear regression analyses with birth year treated as a continuous predictor. Regression coefficients (B), 95% confidence intervals, and model fit indicators are reported. Differences in nutritional status distribution across cohorts were evaluated using chi-square tests, including tests for linear trend across ordered birth cohorts. Statistical significance was set at *p* < 0.05.

The study was based exclusively on anonymized archival administrative records. Access to and use of the archival data for scientific purposes were approved by the Ministry of Defense of Montenegro. All records were digitized and analyzed in anonymized form, and no direct contact with participants occurred. In accordance with national legislation governing the use of historical administrative data for research purposes, individual informed consent was not required.

## Results

Descriptive statistics for body height, body weight, and BMI stratified by birth cohort are presented in Table 1.

**Table 1:** Anthropometric characteristics of male adolescents from Montenegro by birth cohort.

Year of birth (number of subjects)		Mean ± SD			
		Age (years)	Body height (cm)	Body weight (kg)	Body mass Index (kg/m^2^)
1961	(7,866)	18.30 ± 0.71	176.59 ± 6.83	67.79 ± 8.54	21.71 ± 2.20
1962	(10,516)	18.00 ± 0.49	176.80 ± 6.89	67.58 ± 8.53	21.59 ± 2.15
1963	(10,423)	18.06 ± 0.59	176.70 ± 6.88	67.10 ± 8.83	21.46 ± 2.33
1964	(5,363)	17.96 ± 0.57	176.52 ± 6.92	67.21 ± 8.96	21.55 ± 2.48
1965	(9,831)	18.02 ± 0.63	176.04 ± 6.92	67.34 ± 9.08	21.70 ± 2.44
1966	(10,392)	18.09 ± 0.46	177.26 ± 6.89	67.90 ± 8.86	21.59 ± 2.37
1967	(9,737)	18.05 ± 0.42	176.91 ± 6.87	68.16 ± 8.92	21.76 ± 2.41
1968	(9,392)	18.03 ± 0.37	176.80 ± 6.97	68.45 ± 9.13	21.88 ± 2.49
1969	(9,754)	17.99 ± 0.29	177.05 ± 6.95	68.51 ± 9.40	21.84 ± 2.59
Total	(83,274)	18.06 ± 0.52	176.76 ± 6.91	67.80 ± 8.93	21.67 ± 2.39

Statistically significant cohort differences were observed (*p* < 0.001), but effect sizes were negligible and no meaningful secular trends were identified.

The mean body height of the total sample was 176.76 ± 6.91 cm. Slight variations in average height were observed across birth cohorts, with the highest mean value recorded among participants born in 1966 (177.26 ± 6.89 cm) and the lowest mean value among those born in 1965 (176.04 ± 6.92 cm). The mean body weight of the total sample was 67.80 ± 8.93 kg, while the overall mean BMI was 21.67 ± 2.39 kg/m^2^.

One-way ANOVA revealed statistically significant differences across birth cohorts for body height (*F*(8, 83,265) = 24.28, *p* < 0.001), body weight (*F*(8, 83,265) = 31.44, *p* < 0.001), and BMI (*F*(8, 83,265) = 31.82, *p* < 0.001). However, effect sizes were negligible and differences between cohorts were small in absolute terms, indicating that these statistically significant findings were primarily driven by the exceptionally large sample size rather than meaningful secular changes.

Linear regression analyses showed minimal secular changes across successive birth cohorts. A small positive trend was observed for body height (B = 0.049 cm per cohort year, *p* < 0.001), body weight (B = 0.143 kg per cohort year, p < 0.001), and BMI (B = 0.035 kg/m^2^ per cohort year, *p* < 0.001). Nevertheless, the explained variance for all models was extremely low (R^2^ ≤ 0.002), indicating the absence of meaningful secular trends during the observed period.

To assess whether the inclusion of older examinees influenced the findings, a sensitivity analysis was conducted restricting the sample to individuals within the typical examination age range (17–20 years; *n* = 82,083). The results remained unchanged, showing no meaningful secular trends in body height, body weight, or BMI across birth cohorts. Regression coefficients and explained variance were nearly identical to those obtained from the full sample, confirming that the inclusion of a relatively small number of older examinees did not affect the overall conclusions of the study.

Taken together, these findings indicate relative stability of anthropometric characteristics such as body height and body weight, as well as BMI among Montenegrin male adolescents during the study period.

The distribution of nutritional status categories by birth cohort is presented in Table 2.

**Table 2:** The nutritional status of male adolescents from Montenegro by birth cohort.

Year of birth (number of subjects)		Underweight		Normal weight		Overweight		Obesity	
		*N*	%	*N*	%	*N*	%	*N*	%
1961	(7,866)	401	5.1	6,979	88.7	453	5.8	33	0.4
1962	(10,516)	559	5.3	9,330	88.7	593	5.6	34	0.3
1963	(10,423)	755	7.2	9,007	86.4	601	5.8	60	0.6
1964	(5,363)	386	7.2	4,563	85.1	370	6.9	44	0.8
1965	(9,831)	652	6.6	8,338	84.8	779	7.9	62	0.6
1966	(10,392)	684	6.6	8,943	86.1	696	6.7	69	0.7
1967	(9,737)	557	5.7	8,359	85.8	764	7.8	57	0.6
1968	(9,392)	540	5.7	7,926	84.4	850	9.1	76	0.8
1969	(9,754)	671	6.9	8,097	83.0	894	9.2	92	0.9
Total	(83,274)	5,205	6.3	71,542	85.9	6,000	7.2	527	0.6

Differences in nutritional status distribution across birth cohorts were statistically significant (χ^2^(24) = 352.19, *p* < 0.001), with a significant linear trend toward higher prevalence of overweight and obesity in younger cohorts (linear-by-linear association χ^2^ = 91.54, *p* < 0.001).

In the total sample, 6.3% of participants were classified as underweight, 85.9% as normal weight, 7.2% as overweight, and 0.6% as obese. The prevalence of underweight was highest in the 1963 and 1964 cohorts (7.2%) and lowest in the 1961 cohort (5.1%). The highest proportion of normal weight was observed among those born in 1961 and 1962 (88.7%), whereas the lowest proportion was recorded in the 1969 cohort (83.0%).

Chi-square analysis demonstrated significant differences in nutritional status distribution across birth cohorts (χ^2^(24) = 352.19, *p* < 0.001). A significant linear trend toward higher prevalence of overweight and obesity in younger cohorts was also observed (linear-by-linear association χ^2^ = 91.54, *p* < 0.001). Despite statistical significance, absolute differences between cohorts remained modest, with overweight prevalence increasing from 5.8% in the 1961 cohort to 9.2% in the 1969 cohort, while obesity remained below 1% across all cohorts.

## Discussion

The present study provides the most comprehensive nationwide analysis to date of body height, body weight, BMI, and nutritional status among male adolescents in Montenegro during the late 20th century. Based on more than 83,000 standardized medical examinations conducted between 1979 and 1987, the findings substantially reduce a longstanding historical data gap and establish a relevant reference framework for evaluating long-term secular trends in the Montenegrin population.

**Figure 1:**
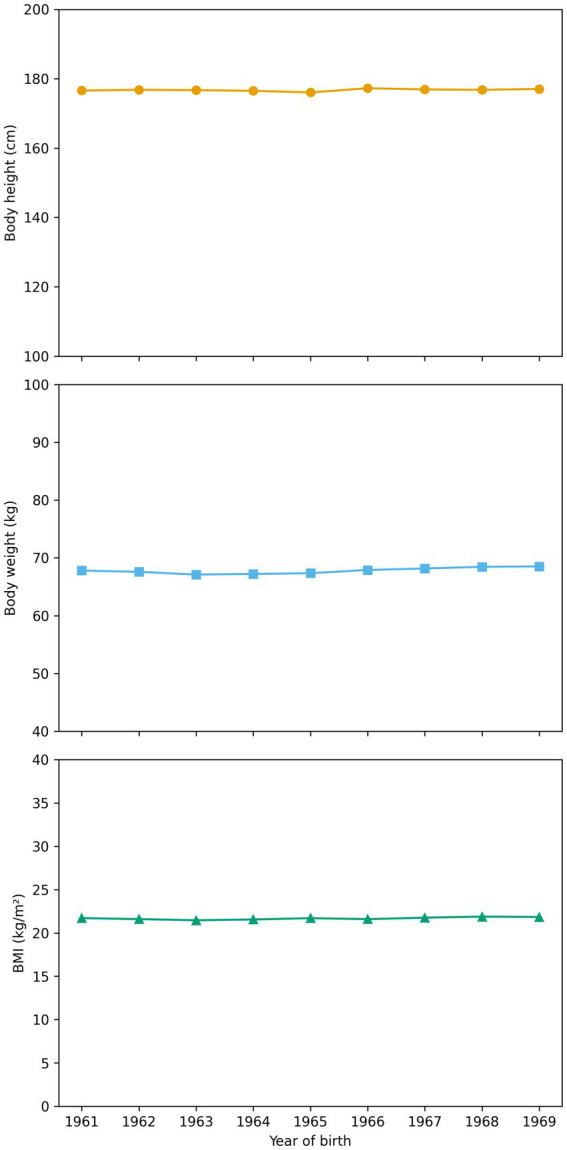
Body height, body weight and BMI across birth cohorts of male adolescents in Montenegro.

The average body height observed in the present study (176.76 ± 6.91 cm) confirms that Montenegrin male adolescents belonged to one of the tallest populations in Europe during the observed period, consistent with findings from both historical and contemporary research in the Dinaric region (7, 8). Although statistically significant differences between birth cohorts were detected, these differences were minimal in absolute terms and associated with negligible effect sizes. Linear regression analyses further indicated only marginal increases across cohorts, with extremely low explained variance. Taken together, these results point to a high degree of stability in body height among Montenegrin male adolescents during the 1980s rather than meaningful secular change. This pattern is consistent with previous local and regional studies conducted in Montenegro for the same period, which likewise reported stable height values across cohorts (14, 15, 17). The relative stability of body height (Figure 1) during the examined period may reflect a temporary plateau following earlier increases in stature observed during the first half of the 20th century. Similar phases of stabilization have been documented in several European populations during periods of socioeconomic consolidation prior to renewed secular increases in later decades (2, 4).

**Figure 2:**
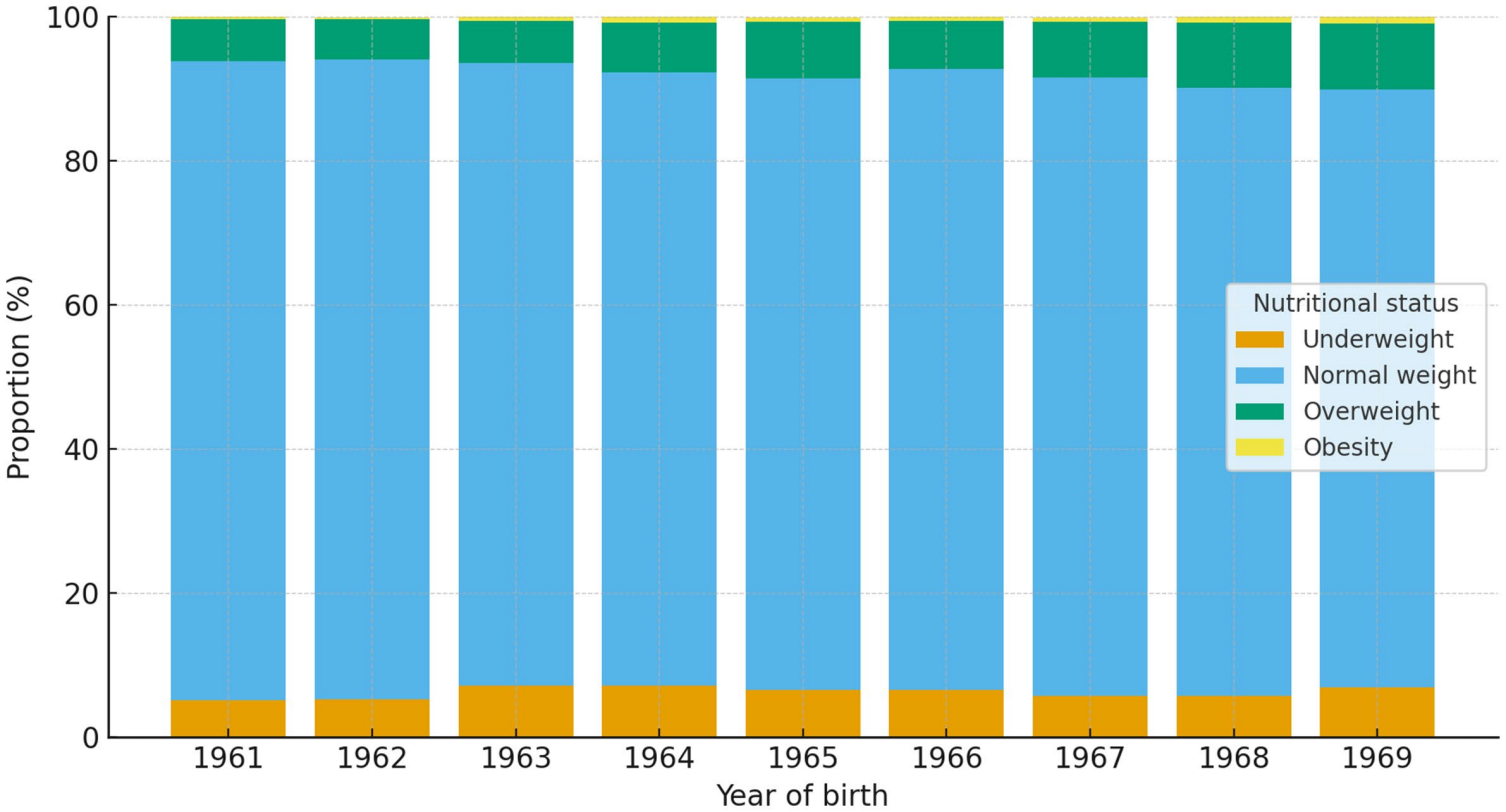
Distribution of nutritional status across birth cohorts of male adolescents in Montenegro.

With regard to body weight and BMI, the results indicate a generally favorable nutritional profile among Montenegrin male adolescents during the study period (Figure 1). More than 85% of participants were classified as having normal BMI, while the prevalence of overweight (7.2%) and obesity (0.6%) remained low compared with contemporary national (11) and global estimates (12, 22). Although statistically significant cohort differences and modest positive trends in body weight and BMI were observed, the magnitude of these changes was small and of limited practical relevance. These findings support the interpretation that the 1980s represented a period characterized by relatively stable and generally favorable nutritional status among male adolescents in Montenegro, preceding the more pronounced increases in overweight and obesity documented in later decades. On the other hand, a gradual increase in the proportion of overweight and obese individuals was observed in younger cohorts, and formal trend analyses confirmed that this pattern was statistically significant (Figure 2). However, the absolute prevalence of obesity remained very low throughout the study period, and the observed changes should be interpreted as early indications of shifts that became more evident in subsequent decades rather than as substantial changes within the examined timeframe.

A major strength of this study lies in its nationwide scope and exceptionally large sample size derived from standardized military medical examinations. The use of archival data covering nearly the entire male adolescent population provides a rare and valuable historical reference for assessing long-term anthropometric and nutritional trends. In contrast to previous studies limited to individual municipalities, the present analysis enables more reliable generalization at the national level. However, several limitations should be also acknowledged. First, the study is based on a retrospective cross-sectional design and includes only male adolescents undergoing mandatory military medical examinations. Although coverage was extensive, it cannot be fully excluded that certain subgroups (e.g., individuals exempted for medical or administrative reasons) were underrepresented. Second, the analysis is limited to late adolescence, and some individuals may not have reached final adult height at the time of examination, although the mean age of approximately 18 years suggests that most participants were close to physical maturity (23). In addition, the inclusion of a relatively small number of older examinees could be considered a potential source of heterogeneity. However, sensitivity analyses restricted to the typical examination age range confirmed that the results remained unchanged, indicating that the overall findings were robust and not influenced by the inclusion of older individuals. Third, the dataset did not include detailed socioeconomic or regional variables, preventing analysis of potential regional disparities or social determinants of anthropometric outcomes. Future research integrating such variables would provide additional insight into the drivers of long-term trends.

Despite these limitations, the present study provides the first comprehensive national overview of anthropometric characteristics and nutritional status among male adolescents in Montenegro during the second part of 20th century. The findings indicate a period of relative stability in body height, body weight, and BMI during the 1980s, accompanied by only modest early increases in overweight prevalence. By establishing a relevant historical baseline based on standardized nationwide archival data, this study offers an essential reference for future research on secular trends and contemporary public health developments in Montenegro and the wider Dinaric region.

## Data Availability

The datasets analyzed in this study are not publicly available due to their archival and administrative nature and restrictions imposed by the Ministry of Defense of Montenegro. Access may be granted upon reasonable request for scientific purposes and with permission from the Ministry of Defense. Requests should be directed to the corresponding author. Requests to access these datasets should be directed to Stevo Popovic, stevop@ucg.ac.me.
